# Genome-wide loss-of-function analysis of deubiquitylating enzymes for zebrafish development

**DOI:** 10.1186/1471-2164-10-637

**Published:** 2009-12-30

**Authors:** William KF Tse, Birgit Eisenhaber, Steven HK Ho, Qimei Ng, Frank Eisenhaber, Yun-Jin Jiang

**Affiliations:** 1 Laboratory of Developmental Signalling and Patterning, Genes and Development Division, Institute of Molecular and Cell Biology, Agency for Science, Technology and Research (A*STAR), 61 Biopolis Drive, 138673 Singapore; 2 Experimental Therapeutics Centre, Agency for Science, Technology and Research (A*STAR), 31 Biopolis Way, 138669 Singapore; 3 Bioinformatics Institute, Agency for Science, Technology and Research (A*STAR), 30 Biopolis Street, 138671 Singapore; 4 Department of Biological Sciences, National University of Singapore, 14 Science Drive, 117543 Singapore; 5 School of Computer Engineering, Nanyang Technological University, 50 Nanyang Drive, 637553 Singapore; 6 Department of Biochemistry, National University of Singapore, 8 Medical Drive, 117597 Singapore; 7 Current address: Laboratory of Physiology, Ocean Research Institute, The University of Tokyo, 1-15-1 Minamidai, Nakano-ku, Tokyo 164-8639, Japan; 8 Current address: Division of Molecular and Genomic Medicine (DMGM), National Health Research Institutes (NHRI), No 35, Keyan Road, Zhunan Town, Miaoli County 35053, Taiwan

## Abstract

**Background:**

Deconjugation of ubiquitin and/or ubiquitin-like modified protein substrates is essential to modulate protein-protein interactions and, thus, signaling processes in cells. Although deubiquitylating (deubiquitinating) enzymes (DUBs) play a key role in this process, however, their function and regulation remain insufficiently understood. The "loss-of-function" phenotype studies can provide important information to elucidate the gene function, and zebrafish is an excellent model for this goal.

**Results:**

From an *in silico *genome-wide search, we found more than 90 putative DUBs encoded in the zebrafish genome belonging to six different subclasses. Out of them, 85 from five classical subclasses have been tested with morpholino (MO) knockdown experiments and 57 of them were found to be important in early development of zebrafish. These DUB morphants resulted in a complex and pleiotropic phenotype that, regardless of gene target, always affected the notochord. Based on the *huC *neuronal marker expression, we grouped them into five sets (groups I to V). Group I DUBs (*otud7b, uchl3 *and *bap1*) appear to be involved in the Notch signaling pathway based on the neuronal hyperplasia, while group IV DUBs (*otud4, usp5, usp15 *and *usp25*) play a critical role in dorsoventral patterning through the BMP pathway.

**Conclusion:**

We have identified an exhaustive list of genes in the zebrafish genome belonging to the five established classes of DUBs. Additionally, we performed the corresponding MO knockdown experiments in zebrafish as well as functional studies for a subset of the predicted DUB genes. The screen results in this work will stimulate functional follow-up studies of potential DUB genes using the zebrafish model system.

## Background

During the past 15 years, protein modification by ubiquitin (UBQ) and ubiquitin-like (UBL) molecules has been suggested to be an important mechanism in regulating numerous critical cellular processes, such as signal transduction, transcriptional control, protein degradation, epigenetic modification and intracellular localization. UBQ is a 76 amino-acid protein that is covalently linked to lysine residues of target proteins in a multi-step process. The process of UBQ conjugation involves three basic classes of enzymes. UBQ-activating enzymes (E1s) activate the C terminus of UBQ, resulting in the formation of an ATP-dependent thio-ester that is linked to the E1's active site - cysteine residue. Afterwards, UBQ is transferred to the active site - cysteine residue - of an E2 conjugating enzyme. The third class of enzymes, the UBQ-protein ligases (E3s) catalyze the final reaction (the UBQ-conjugation process), in which the UBQ is attached to the lysine residue of the protein substrate (reviewed in [1,2]). As a result of the ubiquitylation (ubiquitination) process, the target protein can be modified by a single UBQ (mono-ubiquitylation) or several UBQs (poly-ubiquitylation), which determines the tagging outcome [3].

Deconjugation of UBQ- and/or UBL-modified protein substrates is essential to recycle both the substrate proteins and the UBQ or UBL modifiers; thus, this process influences protein-protein interactions, signaling processes and the pool of free UBQ/UBL within the cell. The deconjugation process is managed by deubiquitylating enzymes (DUBs). The DUBs are in a heterogeneous group of cysteine or metalloproteases [4], which can cleave the scissile peptide bond between the last residue of UBQ (Gly 76) and target proteins. Since their discovery in 1990s, DUBs have been emerging as key regulators in many basic cellular processes. For example, they modulate the ubiquitin-proteasome system (reviewed in [5,6]). Recently, numerous studies have shown that DUBs are linked to diseases such as cancer. CYDL is a tumor suppressor [7-11]; UCHL1 and USP24 are involved in Parkinson's disease [12-16]; ATXN3 influences ataxias [17,18].

Most extensively, DUBs have been studied in the human genome [4]. The classical DUBs can be divided into five subclasses based on their UBQ-protease domains. These are (i) UBQ-specific proteases (USPs), (ii) otubain proteases (OTUs), (iii) UBQ C-terminal hydrolases (UCHs), (iv) Machado-Joseph disease proteases (MJDs) and (v) JAMM motif proteases (JAMMs). Nijman *et al. *have identified 95 DUBs, out of which the overwhelming majority (58) belongs to the USP class. Whereas the DUBs in the JAMM family are metalloproteases, the other four groups belong to the cysteine protease class. Additionally, two more subclasses of DUBs have been suggested based on evidence from sequence homology [19]. The so-called PPPDE group involves papain-like proteases and the WLMs (Wss1p-like proteases) are metalloproteases. Both are hypothesized to play a role in removal of UBQ or UBL from substrate proteins. Experimentally, DUBs can be identified with several approaches such as using hemagglutinin-tagged (HA) UBQ-derived probes [20] or biotin-labeled UBQ aldehyde probes [21] for labeling. These methods had been used to identify several members of the USP, UCH and OTU families [6]. Alternatively, extension of families of known or suspected representatives of DUB classes is possible with the *in silico *collection of families of significantly similar sequences within the homology concept.

It must be noted that the understanding of the DUBs' functions remains very limited despite considerable research efforts. Apparently, the problems are associated with the large number of various DUBs (some of which might compensate for others in parallel pathways) and with their position in the subcellular gene networks where a single DUB can affect several pathways via its protein substrates. One possibility to get new insight into the problem is to involve new model organisms into the analysis and to rely on their specific methodological advantages. The zebrafish (*Danio rerio*) system with its morpholino (MO) technology is superbly suited for easily studying the loss-of-function phenotype *in vivo*, important information necessary to elucidate the genes' function [22,23]. In this work, we use the zebrafish model to identify the MO knockdown phenotypes of potential DUB genes that we have identified with *in silico *methods from the genome sequence. The respective experiments have been carried out for 85 putative DUBs. 57 out of the 85 genes were found to be required for normal development and their morphants have exhibited complex phenotypes always with inclusion of notochord deformations. In order to narrow down the functions in development for some of the genes, we chose a neuronal marker, *huC*, for grouping the DUBs into subgroups that provide hints for specific pathway involvement. This first loss-of-function study of DUB genes in zebrafish provides a valuable resource for further DUB studies in vertebrates.

## Results

### Zebrafish has more than 90 putative DUBs encoded in its genome

DUB-subclass-specific Hidden Markov model and BLAST/PSI-BLAST searches (as described in the Methods section) have been applied for identifying DUBs in the proteome of zebrafish. It should be noted that the first genome-wide search has been started in February 2008 and was regularly repeated (last time, in April 2009). Depending on the zebrafish proteome and the non-redundant protein database, the numbers of hits were slightly different and not all putative DUB sequences were known at the planning stage of experimental work. Therefore, we conclude that our current list of potential DUBs in zebrafish might not be completely exhaustive; yet, it appears comprehensive at the time of writing. For the ubiquitin-specific protease (USP) class containing a UCH (PF0043) domain, we determined 51 proteins. 15 zebrafish proteins resembling OTU-like cysteine proteases (OTUs) have an OTU (PF02338) domain. Another three proteins have the features of Machado-Joseph disease proteases (MJDs) with Josephine domain (PF02099). There are four zebrafish ubiquitin C-terminal hydrolase proteins (UCHs) sharing a Peptidase_C12 (PF1088) domain. The DUB metalloproteases are represented by 14 proteins carrying a Mov34/MPN/PAD-1 family (PF01398) domain (JAMMs). Another four proteins belong to the PPPDE class (PF05903). In total, this results in 91 candidate targets. In Additional file 1, the names and accession numbers of all 91 hits are shown.

The sequence domain architectures of all 91 DUB hits are also shown in Additional file 1. The combination of UBQ-protease domains with domains and motifs from the ubiquitylation pathway (such as ubiquitin-associated UBA, ubiquitin UBQ and ubiquitin-interacting motif, UIM) is not a real surprise, whereas the association with nucleic-acid binding domains (Myb_DNA_binding, PRO8 and Tudor) and protein-binding domains (SWIRM and MATH) would make sense in the context of the involvement of DUB proteins in the regulation of gene expression. The PRPF8 (pre-mRNA-processing-splicing factor 8) protein, a member of the JAMM protein family, is known to be one of the largest and most highly conserved nuclear protein that occupies a central position in the catalytic core of the spliceosome [24]. Another member of the JAMM protein family, MYSM1, is a histone H2A deubiquitinase that regulates transcription by coordinating histone acetylation and deubiquitylation, and destabilizing the association of linker histone H1 with nucleosomes [25]. We searched the scientific literature extensively for evidence for DUB functions and the summary of known DUBs is shown in Additional file 2.

We think that both the human and zebrafish DUB lists are not complete at present and revisions of the genome build or the proteome lists will lead to some correction. As a general trend, the total numbers as well as the numbers of DUBs in the subclasses are quite comparable between both organisms [4]. For the overwhelming majority (about four fifths), there are also clear orthologues in fish and humans (these genes carry the names of their human counterparts in Additional file 1); yet, there are also a few cases of missing equivalents in either organism or organism-specific duplications and it is not clear at present whether genome revisions will resolve these discrepancies. So far, it appears too early to draw biological conclusions out of differences in the DUB lists.

### 57 DUB genes are required for zebrafish early development

At the time of starting the MO experiments, our list comprised 85 gene entries. For most of them, we found records in GenBank and/or the Ensembl genome database (Additional file 3) suitable for straightforward MO design. It is a common and general practice to design the MOs that target the ATG translation sites. Translation-blocking MO interferes with the protein translation process of the target gene. However, ATG translation start site information was missing for 11 sequences and we used a workaround as described in the Methods section. For these genes, we designed MOs targeting splicing of exon-intron junction that is on or in the vicinity of conserved domains (marked in bold italics in Additional file 3).

Among all 85 MOs tested, 26 MOs (31%) led to no detectable morphological phenotypes, while two MOs (2%) resulted in cell death in early developmental stages (cell arrest after 30% epiboly). The remaining 57 MOs (67%) gave different observable phenotypes, including abnormal development in head, brain, eyes, body axis, notochord, precardial region, yolk and tail. Most of the MOs did not specially affect one region but several. The notochord was always affected in the MO knockdown experiments. The results are summarized in Additional file 4. In this table, brief phenotypic descriptions and images of the morphants are shown.

### Classifying DUB genes into five subgroups according to their huC expression level and pattern

**Figure 1:**
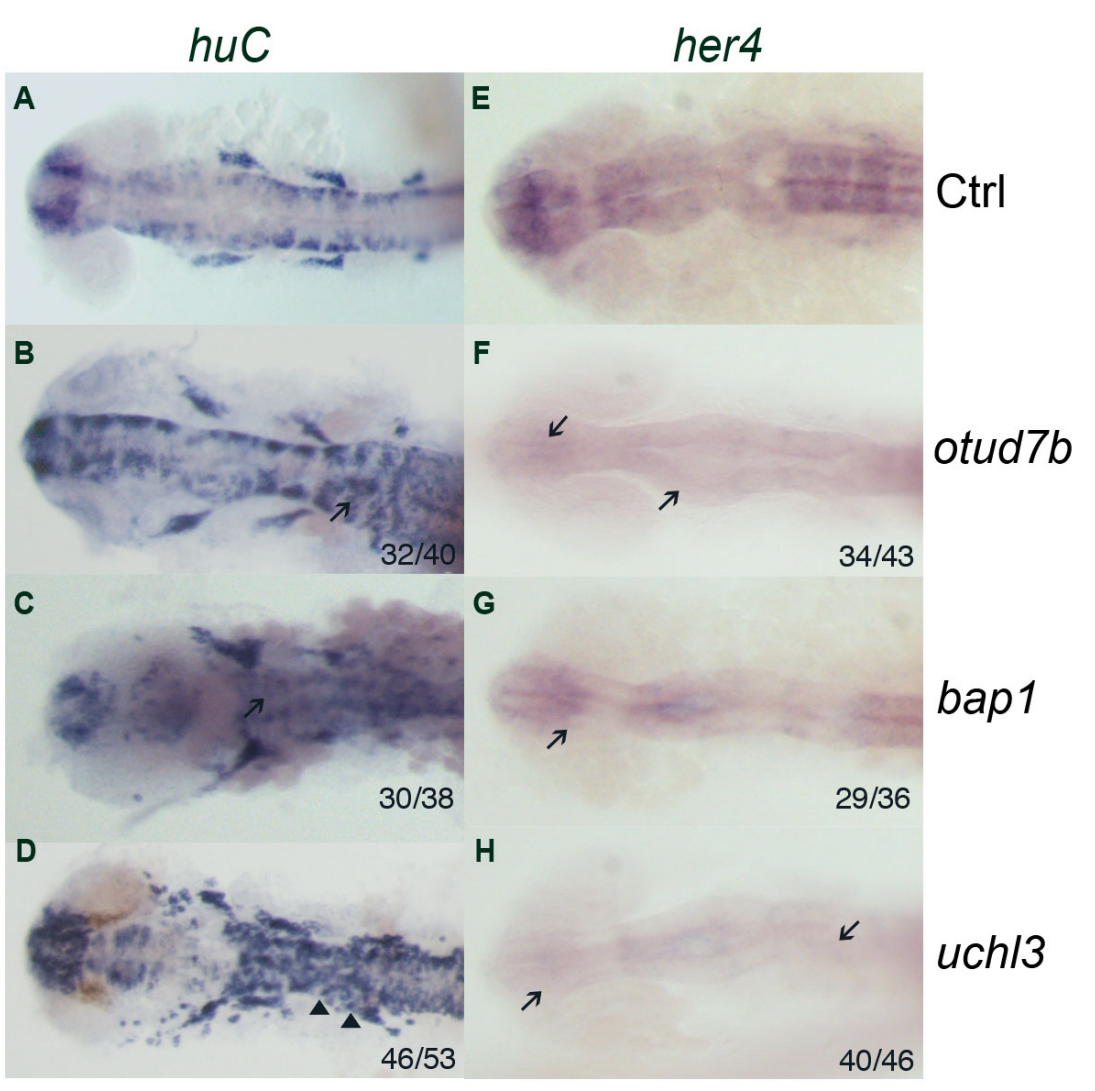
***huC *and *her4 *expression in morphants of group I zebrafish DUBs at 24 hpf**. *huC *and *her4 *expression at 24 hpf, dorsal view. Increase in *huC *expression was found in morphants of group I DUBs when compared to control (A-D). There are 3 members in this group, which named as *otud7b, bap1 *and *uchl3*. The arrows mark the increased *huC *expression area (B-C), while the arrowheads mark the denser expression pattern (D). *uchl3 *morphants showed the most severe effect with both stronger and denser *huC *expression. *her4 *is a downstream target gene in the Notch signaling pathway, increase of neurons may be due to the decrease of the Notch activation. Decrease in *her4 *expression was found in morphants of all group I DUBs when compared to control (E): *otud7b *(F), *bap1 *(G) and *uchl3 *(H). Arrows show the absence of *her4 *expression in the morphants. The number of embryos with the presented phenotype is shown in the right bottom corner of each panel.

**Figure 2:**
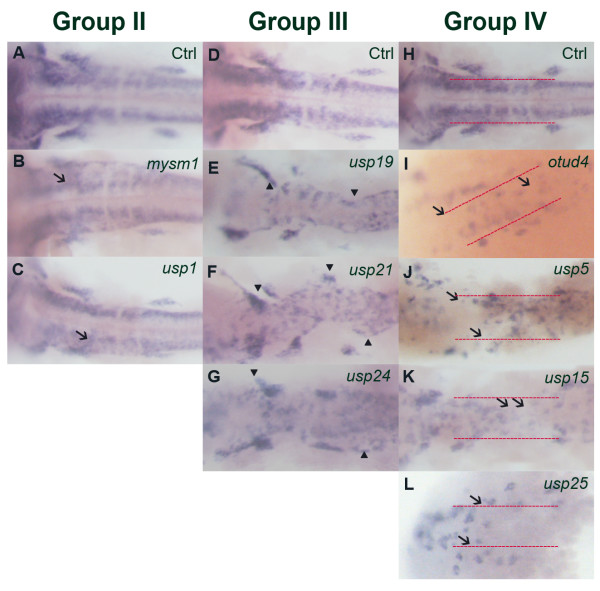
**Comparison of *huC *expression in morphants of groups II, III and IV zebrafish DUBs at 24 hpf**. *huC *expression at 24 hpf, dorsal view. Decreased *huC *expression was found in morphants of these 3 groups. Group II has 16 members that showed a typical decreased *huC *expression (arrows). Group III has 13 members that showed the decreased *huC *expression with destructive patterning. Lastly, group IV has 4 members that had the most severe decreased and destructive *huC *expression. All 4 members named *otud4, usp5, usp15 *and *usp25 *could not form a normal neuronal pattern in the head region. Arrowheads mark the abnormal patterns in morphants of group III; while arrows indicate the absence of neurons in morphants of group IV.

As we mentioned above, most of the DUB genes gave pleiotropic phenotypes. It appears difficult to classify the diverse phenotypes based on their varied morphology. Therefore, we applied additional *in situ *staining of the neuronal *huC *marker. We chose *huC *as the primary marker because of its strong and easily recognizable staining patterns in zebrafish early development. In addition, neuronal development is always a hot topic and research-focusing point. In the screen, its distinctive distribution patterns allowed classifying 83 out of 85 DUB genes into five groups (except for *zranb1a *and *usp46 *(2%) that resulted in cell death). The largest group includes 47 members (group V, 55%), which shows no change in *huC *expression level or patterning. Within this group, all the members exhibit the same neuronal pattern as the control. Neurons are formed in a well-organized manner (data not shown). Group I is the only group that has an increased *huC *expression (increase in both expression level and number of neurons) and/or enlarged patterning and is composed of three members (4%). Overgrown hindbrain neurons were found among them (Figure 1A-D). Groups II to IV are characterized by a decrease in *huC *expression with different patterns. Group II shows decreased *huC *expression without changed patterning and comprises 16 members (19%). Group III exhibits decreased *huC *expression with slightly destructive patterning effect and has 13 members (15%). They have abnormal shape of hindbrain neurons, such as curved neuron pattern and uneven cranial ganglia length. Finally, group IV features a decrease of *huC *expression together with severely destructed patterning and it is made up of 4 members (5%). All the members in this group cannot form organized neuronal patterns. Besides, individual small clusters of neurons were observed. Moreover, all the cranial ganglia structures were lost in this group. Selected results among them are shown in Figure 2. Since groups I and IV showed distinct *huC in situ *results, we next focused on these two groups for general signaling and developmental studies. The classification is summarized in Additional file 4.

### *otud7b*, *bap1 *and *uchl3 *(group I) may be involved in Notch signaling

The knockdown of group I DUB genes resulted in increased *huC *expression (Figure 1A-D, Additional file 5), indicating an increase in neurons. Neurogenesis is the process of forming neurons in the early development. Neuronal death during the development is a normal process happened in the normal development [26]. One of the well known midline cell fate signaling is the Notch pathway [27,28]. Inactivation of Notch pathway leads to a failure of the lateral inhibition, which results in premature differentiation of neural progenitors and permits neuron mass production.

Activation of Notch leads to *her4 *expression [29]. Morphants of all group I DUB genes (*otud7b*, *bap1 *and *uchl3*) showed neuronal hyperplasia, indicating a compromise in Notch-dependent lateral inhibition during development (Figure 1A-D). Consistently, all their morphants showed decreased *her4 *expression in descending order of *otud7b*, *bap1 *and *uchl3 *(Figure 1E-H, Additional file 5), indicating that their corresponding genes may be involved in the Notch signaling pathway.

### *otud4*, *usp5*, *usp15 *and *usp25 *(group IV) play critical roles in dorsoventral patterning

From the *huC *expression, we noticed that neurons of group IV morphants are not well organized, implying that their dorsoventral patterning might be affected in the early stages. From the phenotypic studies at 24 hpf, the group IV morphants have dorsalized phenotypes that ranged from C1 to C5 (Additional file 6), where C5 shows the strongest dorsalized phenotype [30,31]. Their phenotypes became milder afterwards (Additional file 4), which might be due to the decreasing MO blocking effect at later stages and/or negative feedback regulation on gene expression [32]. We collected the MO-injected embryos for *in situ *staining at about 50%-60% epiboly and 10-somite stages. Several molecular markers were used to examine the mesoderm, ectoderm and dorsolateral regions.

**Figure 3:**
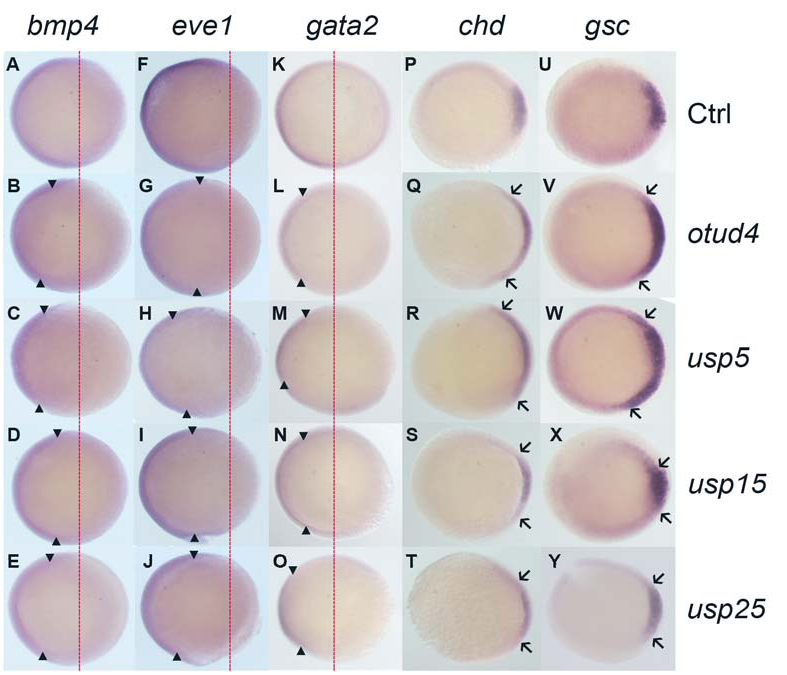
**Comparison of ventral (*bmp4, eve1, gata2*) and dorsal (*chd, gsc*) markers expression in morphants of group IV zebrafish DUBs at 50-60% epiboly**. Ventral markers (*bmp4*, *eve1 *and *gata2*) and dorsal markers (*chd *and *gsc*) expression of group IV morphants at 50-60% epiboly stage, animal pole views, dorsal towards the right. *otud4 *(B, G, L, Q, V), *usp5 *(C, H, M, R, W) and *usp25 *(E, J, O, T, Y) morphants showed narrower expression pattern for ventral markers (A-O) but wider expression pattern of dorsal markers (P-Y). *usp15 *showed a similar expression pattern (D, I, N, S, X) with control (A, F, K, P, U). Red dot lines indicate the normal expression margin of ventral markers in wild-type embryos.

Three different markers of ventral territories (*bmp4*, *eve1 *and *gata2*) and two dorsal ectoderm and mesoderm markers (*chd *and *gsc*) were used. Bone morphogenetic protein 4 (Bmp4) is important to the dorsoventral patterning of the mesoderm. The failure of its expression in dorsal blastomeres causes the ventralization of the embryo [33-35]. *eve1 *is a zebrafish homeobox gene similar to *even-skipped *in *Drosophila *[36]. It is strongly expressed in the ventrolateral marginal cells. *gata2 *is a hematopoietic transcription factor gene [37] that is expressed in a pattern similar to *eve1*. It is a marker for ventral ectoderm and hematopoietic cells in ventral mesoderm. All three markers showed similar results in the group IV morphants. Their transcripts in the ventral half of the marginal and the animal zone are expressed in a more restricted area than controls (Figure 3A-O, Additional file 7). For the dorsal patterning, we used *chordin *(*chd*) and *goosecoid *(*gsc*). Chordin can antagonize and repress BMP4 and is normally dorsally restricted [38]. Similarly, *gsc *expression is also restricted to dorsal region [39]. Expression of *chd *and *gsc *in the morphants was expanded into lateral domains (Figure 3P-Y). To summarize, the ventral fates were affected and restricted in a smaller area while the dorsal region was expanded during gastrula.

**Figure 4:**
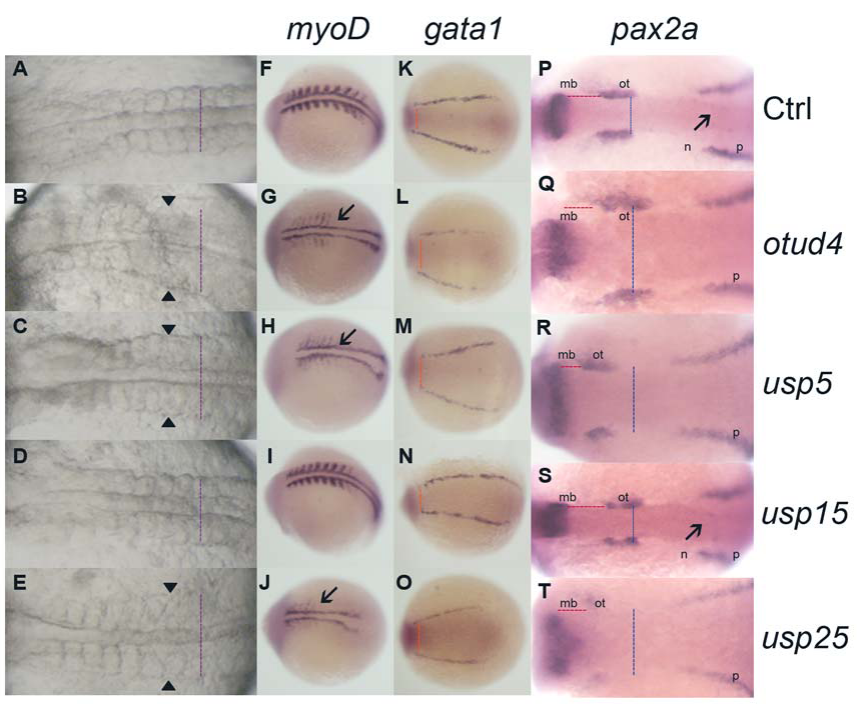
**Comparison of *myoD, gata1 *and *pax2a *expression in morphants of group IV zebrafish DUBs at 10-somite stage**. Morphology of group IV morphants at 10-somite stage (A-E). Lateral expansion of somite muscles were indicated (arrowheads and purple dot lines) in *otud4 *(B), *usp5 *(C) and *usp25 *(E) morphants. *usp15 *(D) morphants showed a similar phenotype as control (A). *myoD*, *gata1 and pax2a *expression at 10-somite stage, dorsal view (F-T). Orange dot lines represent the distance between two lateral stripes; blue dot lines indicate the distance between two optic vesicles; while red dot lines show the distance between midbrain and otic vesicle. mb, midbrain; ot, otic vesicle; n, neuronal and p, pronephric precursor expression domains. *otud4, usp5 *and *usp25 *morphants showed features of dorsalization: lateral expression of *myoD *(G-J, marked with arrows), *pax2a *(Q-T) and widening of the *gata1 *(L-O) mesoderm distance expression. *usp15 *(I, N, S) showed a similar expression to control (F, K, P). All are head to the left.

The shift of various marker genes (*bmp4*, *chd*, *eve1*, *gata2 *and *gsc*) would result in phenotypic changes in the later developmental stages. In order to confirm that the dorsalized effects are maintained after gastrulation, we chose some other *in situ *markers for the studies at 10-somite stage: *myoD *is used for dorsal mesoderm and somite muscle staining [40,41]; *gata1 *is ventrally expressed in presumptive hematopoietic cells in two lateral stripes [37,42]; and *pax2a *is used for marking the presumptive neural region [43]. From the *in situ *results, we found that *myoD *and *pax2a *expression patterns were expanded in group IV morphants, indicating a dorsalized phenotype [41,44]. *myoD *expression in *otud4*, *usp5 *and *usp25 *morphants was weaker but wider. The number of stained somatic muscles was decreased. *usp25 *morphants showed the most severe result while *usp15 *showed a similar *myoD *pattern to wild-type (Figure 4F-J). For *gata1 *staining, widening of the two lateral stripes of presumptive hematopoietic cells was found in *otud4*, *usp5 *and *usp25 *morphants. Same as the results of other probes, *usp15 *morphants showed a similar *gata1 *expression pattern as wild-type (Figure 4K-O). Similarly, in *pax2a in situ *staining, *usp15 *morphants showed a similar expression pattern as wild-type. In *otud4*, *usp5 *and *usp25 *morphants, distance between two otic vesicles was increased, which featured dorsalization. In addition, *usp25 *morphants also showed a decreased *pax2a *expression in otic vesicles (Figure 4P-T). Taken together, the abnormal dorsoventral patterning in morphants of *otud4, usp5, usp15 *and *usp25 *genes started from gastrula and continued in later developmental stages. In addition, while *usp25 *morphants always showed a more severe dorsalized pattern, *usp15 *always showed the least.

### The group IV DUB genes are likely involved in the BMP pathway

**Figure 5:**
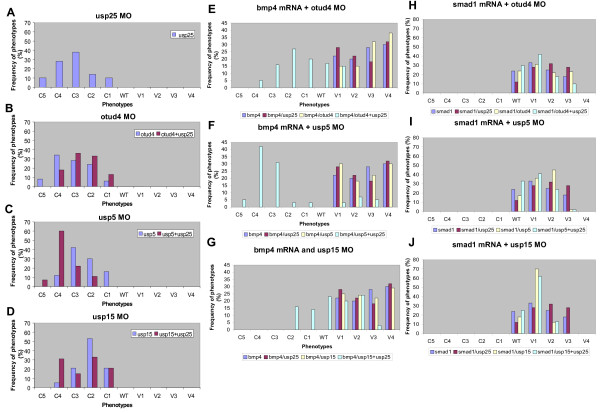
**Frequency of dorsalized and ventralized phenotypes after knockdown of group IV DUB genes and overexpression of Bmp signaling genes**. Panels A to D showed the frequency of dorsalized phenotypes after knockdown of group IV DUB genes. Noted that severe dorsalized phenotypes were found after double-knockdown of *usp5+usp25 *or *usp15+usp25 *(C, D). Panels E to G showed the phenotypes' frequencies after overexpression of *bmp4 *and group IV DUB genes knockdown. Noted that there were phenotypic shift from ventralization to dorsalization after double knockdown of two DUB genes, with more obvious changes in *otud4+usp25 *(E) and *usp5+usp25 *(F) double knockdown. Panels H to J showed the results of the *smad1 *overexpression and double knockdown of group IV DUB genes. Noted that the ventralized effects were maintained in the double knockdown of group IV DUB genes. Collectively, the results suggested that group IV DUB genes are involved in the BMP pathway and function between Bmp4 and Smad1. Noted that there were three different Y axis scales in the figure (A-D; E-G; H-J). The number of injected embryos was showed in Table 1.

**Figure 6:**
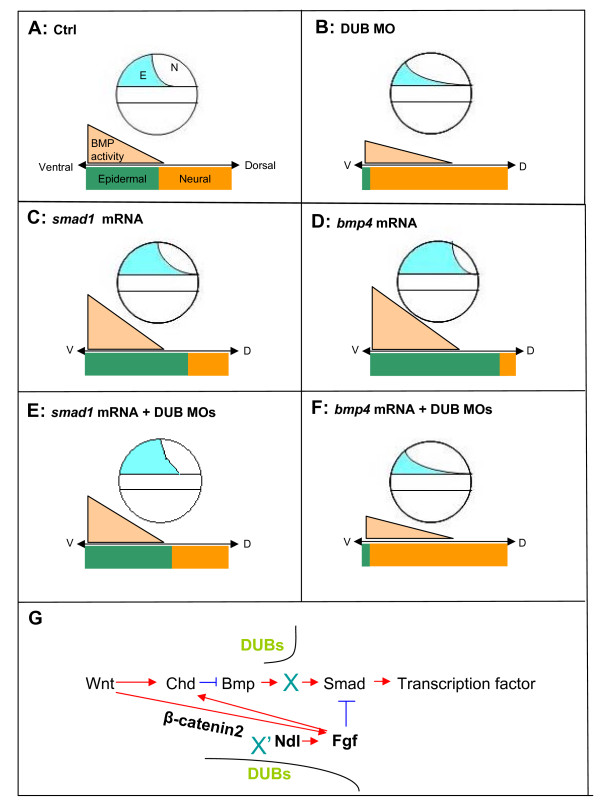
**Proposed model of group IV DUB genes in the BMP pathway**. Group IV DUB genes (*otud4, usp5, usp15 *and *usp25*) are involved in the BMP pathway. Panel A shows the normal wild type embryo with usual BMP activity. Panel B shows that single group IV MO knockdown results in dorsalized phenotypes. Panel C shows that overexpression of *smad1 *leads to ventralized phenotypes (V1-V3). Panel D shows that *bmp4 *overexpression results in severe ventralized phenotypes (V3, V4). Panel E shows that co-injection of combination of group IV MOs and *smad1 *mRNA results in mild ventralized phenotypes. Panel F shows that co-injection of combination group IV MOs and *bmp4 *mRNA causes severe dorsalized phenotypes as in panel B. Double knockdown of group IV MOs with *bmp4 *or *smad1 *overexpression causes different results. Overexpression of *bmp4 *could not rescue the dorsalized phenotypes that caused by double MOs knockdown, while *smad1 *could. This implies that group IV DUBs function between Bmp4 and Smad1 in the BMP pathway. Since co-injection experiments could not result in absolute dorsalized or ventralized phenotypes, which suggests that the DUB genes might interact with other dorsoventral signaling pathways. The proposed working model is shown in G. Taken together with the real-time results (Additional file 9), the group IV MO will increase the mRNA expression of different signaling genes (bolded), which results in stimulating Chd and inhibiting Smad. Collectively, all the effects will inhibit the BMP pathway and cause dorsalized effect. We propose that group IV DUBs will act on the substance X that is directly involved in the BMP pathway. Besides, they may interact with another common substrate X' that stimulates different signaling genes and generates secondary dorsal-ventral effects in the zebrafish early development.

The BMP signaling pathway is well known for its essentiality in many developmental processes [45]. In zebrafish, it plays a main role in determining the ventral cell fates [46]. Briefly, the BMP signaling pathway starts from the BMP receptors activation. The receptors are mediated by BMPs and will active the Smad proteins (R-Smad) through phosphorylation. The Smad protein will then bind to a common mediator Smad (Co-Smad) to form complexes, which will finally be translocated into the nucleus and act as transcriptional regulators. Mutations of ventral *bmp *gene will result in the dorsalization of the embryo. *swirl *(*swr*) and *snailhouse *(*snh*) [30] are two well known mutants that have mutations in *bmp2b *[31] and *bmp7 *genes [47,48], respectively. To gain further insights into where group IV DUBs function in the BMP pathway, we preformed co-injection of group IV MOs and selected mRNA of BMP signaling genes. Single mRNA injections of *bmp4 *and *smad1 *resulted in ventralized phenotypes as previously described [31,49]. When embryos were injected with *bmp4 *mRNA and group IV MO, we observed ventralized phenotypes (Figure 5E-G). The results seemed to disagree with our hypothesis, since Bmp4 is at the uppermost position in the BMP pathway. However, because DUBs are enzymes, their functions might be compensated or replaced by others. Thus, we preformed co-injection of *bmp4 *mRNA with different combination of group IV DUB MOs. Based on the single MO injection result, we chose the most effective *usp25 *MO as a basic component and all the MO concentrations were halved as single injection. Except for the mild effect of *usp15+usp25 *double knockdown, both *otud4+usp25 *and *usp5+usp25 *MOs significantly enhanced the dorsalized phenotypes in embryos co-injected with *bmp4 *mRNA (Figure 5E-G). For co-injection of *smad1 *mRNA and group IV MOs, ventralized phenotypes were found (Figure 5H-J). Specific injection amounts were tabulated in Table 1. In the table, we introduced the Dorsal-Ventral (DV) value, which was calculated by the frequencies of phenotypes times a fixed value (5 for C5, 4 for C4, -4 for V4 and -3 for V3 phenotypes, etc.). The final DV value indicated the average result of the co-injection experiments. To summarize, the combination MOs could not rescue ventralized phenotypes caused by *smad1 *mRNA injection (negative DV value), while maintained their dorsalized phenotypes when co-injected with the *bmp4 *mRNA (positive DV value except the mild effect of *usp15*+*usp25*). These data suggested that the group IV DUBs act downstream of Bmp4 and upstream of Smad1 in dorsoventral patterning. The substrate of the particular E3 ligase in the BMP pathway can be deubiquitylated more efficiently by two DUBs. Collectively, all the group IV DUB genes are involved in the BMP pathway and can affect the early dorsoventral patterning in the zebrafish. The proposed working model is depicted in Figure 6A-G. We classified the phenotypes based on Additional file 6 and further confirmed by *in situ *staining using different molecular markers at different stages (Additional file 8).

**Table 1:** Dorsal-ventral value of RNA and/or MO injection studies

RNA/MO	pg/em	pmol	n ep	n	DV value
*-/otud4*		0.5	3	104	3.14
*-/usp5*		2.0	3	101	2.50
*-/usp15*		0.5	3	127	2.10
*-/usp25*		2.0	3	185	3.14
*-/otud4+usp25*		0.25/1.0	2	100	2.59
*-/usp5+usp25*		1.0/1.0	2	110	3.63
*-/usp15+usp25*		0.25/1.0	2	78	2.56

*bmp4/-*	2.5		3	98	**-2.66**
*bmp4/otud4*	2.5	0.5	3	71	**-2.93**
*bmp4/usp5*	2.5	2.0	3	65	**-2.52**
*bmp4/usp15*	2.5	0.5	3	78	**-2.55**
*bmp4/usp25*	2.5	2.0	3	82	**-2.54**
*bmp4/otud4+usp25*	2.5	0.25/1.0	2	93	1.27
*bmp4/usp5+usp25*	2.5	1.0/1.0	2	118	2.63
*bmp4/usp15+usp25*	2.5	0.25/1.0	2	132	**-0.31**

*smad1/-*	800		3	96	**-1.37**
*smad1/otud4*	800	0.5	3	103	**-1.44**
*smad1/usp5*	800	2.0	3	111	**-1.32**
*smad1/usp15*	800	0.5	3	104	**-0.94**
*smad1/usp25*	800	2.0	3	98	**-1.76**
*smad1/otud4+usp25*	800	0.25/1.0	2	68	**-1.08**
*smad1/usp5+usp25*	800	1.0/1.0	2	71	**-0.95**
*smad1/usp15+usp25*	800	0.25/1.0	2	75	**-0.88**

Two mRNA (*bmp4 *and *smad1*) were co-injected with the group IV MOs (*otud4, usp5, usp15 *and *usp25*). The dorsal-ventral values were calculated as follows: Frequency of phenotypes * [C5: 5; C4: 4; C3: 3; C2: 2; C1: 1; Wt: 0; V1: -1; V2: -2; V3: -3; V4: -4]. The highest DV value is 5, which indicates 100% C5 phenotypes, while lowest is -4, which represents 100% V4 phenotypes. Negative values (ventralized phenotypes) were shown in bold.

Abbreviations: n ep, number of experiments; n, number of scored embryos; DV value, dorsal-ventral value.

## Discussion

In this paper, we describe our identification of candidate DUB genes in zebrafish and the results of the "loss-of-function" screen based on the morpholino technology; thus, vastly extending the available biological information about DUB gene function and creating a resource for their further functional study. To our knowledge, "loss-of-function" studies of DUBs in any kinds of organism have been carried out only for very few genes due to the considerable cost associated with raising genetic knockout- and/or knockdown-modulated animals. Zebrafish is a model organism that is well suited to fill this gap. It is well known for its fast life cycle and the transparency of its embryos. Recently, the techniques of using MO knockdown and mRNA overexpression by microinjection further advance the versatility of using zebrafish as a model system. Developmental processes are always an interesting area for studying gene functions. Thus, we used the zebrafish to perform the DUB genes "loss-of-function" screen to understand their developmental functions.

### More than 90 DUB candidates in zebrafish were found

To begin with, we started an *in silico *screen aimed at identifying potential DUB genes from the zebrafish genome. Although both the genome and the proteome of zebrafish are subject to some changes and corrections in the future, the available sequence data is sufficient for deriving the overwhelming part of the complete genes of DUB candidates. For six of the described seven classes of DUBs (USP, OTU, UCH, MJD, JAMM, PPPDE), we found 91 candidates. As expected, there are no candidates for the WLM group. Both in total number and in respect to the distribution among subclasses, zebrafish and human are not very different. For most (ca. 80%) of the candidates, there are obvious orthologues in both organisms although there are occasional organism-specific duplications or missing DUBs (Table 2). The sequence domain architecture analysis of the DUBs showed co-occurrence with domains characteristic for UBQ/UBL pathways as well as for gene expression, chromatin and cytoskeletal re-structuring processes; thus, it is likely that DUBs influence the respective biomolecular mechanisms (Additional file 1).

**Table 2:** Comparison of DUB genes in human and zebrafish

DUB Family Name	Extra DUB Genes in Zebrafish	Missing DUB Genes (Protein) in Zebrafish	Subtotal in Zebrafish	Subtotal in Human
**USP**	*cyldb*	USP6	**51**	**58**
	*usp2b*	USP9X		
	*usp9*	USP9Y		
	*usp12b*	USP17		
	*usp53*	USP26		
	*usp54b*	USP29		
	*unclassified (1)*	USP35		
	*unclassified (2)*	USP41		
	*unclassified (3)*	USP50		
		USP51		
		USP52		
		USP55		
		#59 (DUB3)		
		#60		
		#61 (TL132-l)		
		#62 (TL132)		

**OTU**	*otud1l*	OTUD6A	**15**	**14**
	*otud3*	OTUBQ2		
	*otud5b*	OTUD1		
	*zranb1b*			

**UCH**			**4**	**4**

**MJD**		ATXN3L	**3**	**5**
		JOS3		

**JAMM**	*eif3hb*	#89 (IFP38)	**14**	**14**
	*stambpb*	#91		

**PPPDE**	*pppde1*		**4**	**n.a.**
	*loc794838*			
	*pppde2a*			
	*pppde2b*			

Through the *in silico *screen, 91 putative DUB genes were found in zebrafish, which were classified into 6 families. Details were summarized in the table. For the first 5 families (USP, OTU, UCH, MJD and JAMM), gene and protein names in human were based on Nijman *et al.*, 2005 [4]. The human homologues of the PPPDE family were not studied in this work. n.a. stands for "not analyzed".

### DUB genes play different roles in the developmental processes

Out of 85 putative zebrafish DUB genes that were subjected to a MO test, 57 DUB morphants show pleiotropic phenotypes throughout the body. From the results, we infer that DUB genes play different roles in the early zebrafish developmental processes, ranging from the dorsoventral patterning during the gastrulation to the fine-tuning of neurons and fin phenotypes. Most of the DUB genes appear multi-functional: they affect not only one and single developmental process. Taking *Cyld *as an example, there are published papers using the *Cyld *knockout mice for the studies. They have different effects on spermatogenesis and osteoclastogenesis [50,51]. Deubiquitylation is an important process for cells to maintain the cellular level of UBQ. Different cellular processes require ubiquitylation. E3 ligase is an enzyme that is involved in the last step of ubiquitylation. E3 ligases like BIR repeat-containing Ubiquitin-conjugating enzyme (BRUCE) and Mind bomb (Mib) perform different functions. BRUCE can regulate the apoptosis and cytokinesis [52,53], while Mib can facilitate the internalization of cell surface Delta in signal-sending cell and promote Notch activation in signal-receiving cells [54,55]. The number of UBQ E3 ligases is far greater than that of DUB enzymes, which implies that, as a trend, one DUB enzyme could deubiquitylate the substrates of several E3 ligases [4]. This fact may explain why our DUB-morphants did not show any unique and specific phenotypes. The resulting normal phenotype in some morphants may be due to the non-specific or/and low dosage of the MOs. Besides, we could not exclude the possibility that some other DUBs could compensate the deubiquitylation process of the knockdown DUB gene. Thus, those 26 DUB genes that did not give phenotypes could be regarded as the "non-essential" DUB genes, while those 59 (including two that are required for survival) DUB genes caused abnormal phenotypes are critical for development.

### Notch signaling and the group I DUB genes

In the vertebrate neuronal development, formation of excess number of neurons during the neurogenesis will decay through apoptosis to ensure the formation of correct neurons' connection and pattern [56]. Neuronal death is a normal process happens during development [26]. Thus, it is abnormal that there are an increased number of neurons in the developmental process. The selection of the neuronal cell death and/or survival is mainly based on various neurotrophic factors and their related signaling pathway [57-59]. Notch signaling is well known for its control of cell fate and regulation of pattern formation [60]. Notch is a transmembrane receptor that is involved in the Notch signaling pathway, which was first identified in the mutant fly with "notches" in the wing [61]. Reports had shown that Notch signaling restricts neural differentiation by inhibiting the expression of proneural genes and its deficiency results in neurogenic phenotypes [62,63]. Our *huC in situ *screen identified three group I DUB genes, whose morphants exhibit an increase of neurons, indicating a premature differentiation - a possible consequence of a failure in the Notch-dependent lateral inhibition. Within these morphants, we further confirmed that the expression level of one of the Notch signaling downstream target gene, *her4*, was decreased. These results suggested that group I DUB genes affected Notch signaling directly or indirectly.

### BMP signaling and the group IV DUB genes

In the *huC *screen, there were four DUB genes whose morphants had severely destructive neuronal patterning. One of the reasons to cause this could be the early defect in dorsoventral patterning. We tested different dorsal and ventral molecular markers at different time points and the results suggested that these morphants are dorsalized. One of the well known pathways involved in the dorsoventral patterning is the BMP pathway, which is required to specify the ventral cell fate [46]. We further underwent the co-injections experiments of mRNA of BMP signaling genes and the DUB MOs to further determine whether these DUBs are involved in the BMP pathway. When we co-injected *smad1 *mRNA and group IV DUB MOs into embryos, the dorsalized phenotypes caused by MO injection alone was rescued, indicating that group IV DUB genes are involved in the BMP pathway. Surprisingly, *bmp4 *mRNA injection with single group IV MO also resulted in ventralized phenotypes, which suggested that their functions might be compensated by others. Thus, we decided to make a combination of MOs among the group IV DUBs. After double knockdown with *bmp4 *overexpression, results showed that there was a shift from V3-V4 phenotypes to dorsalized phenotypes. It is notable that all the experiments could not completely transform the overexpression (ventralized) or knockdown (dorsalized) effects. This observation suggests that group IV DUBs may not simply be involved in the BMP pathway, but also involved in other dorsoventral pathways such as Nodal, Fgf, and Wnt. Reports have shown that these pathways can interact with each others [64-66]. We tested different signaling genes mRNA expressions by the real-time PCR. Consistently, results showed that the group IV MO could affect the signaling genes expression slightly but significantly in Nodal, Fgf and Wnt pathways. Details were shown in Additional file 9. It is not surprising that DUBs are involved and function differently in more than one pathway. Many cellular processes involve ubiquitylation, which suggests that one DUB may deubiquitylate more than one substrate. Here, we only showed the knockdown of group IV DUB genes will change the mRNA expression of different signaling genes; however, we did not know exactly how they are regulated and what is the mechanistic details between group IV DUB genes and them. Further biochemical studies have to be conducted in order to fully understand the nature of the DUBs.

## Conclusion

From the *in silico *screen, we have identified 91 zebrafish DUB candidate genes belonging to six families. Out of the 85 targets that were subjected to a MO knockdown test: two were found to cause cell death and 57 resulted in pleiotropic developmental phenotypes; the remaining 26 did not show a detectable phenotype. Based on the expression level and pattern changes of *huC*, the 57 cases are classified into five groups. Further functional analyses of group I DUB genes suggest that three DUB genes (*uchl3, otud7b *and *bap1*) are the candidate genes to be functionally associated with the Notch pathway. Group IV DUB genes (*otud4, usp5, usp15 *and *usp25*) appear to be involved in the BMP pathway. Besides, our results suggest that substrates of the E3 ligase in BMP pathway are deubiquitylated more efficiently by two DUBs. To conclude, this paper provides a basic frame for the functional studies of DUB genes and acts as a screening step for researchers to pick up their specific DUB genes for follow-up studies based on the phenotypes. We understand that the screen might not be fully complete and that further research and in-depth studies are required. By releasing the genetic and functional information at this stage, the research efficiency with respect to zebrafish DUB genes will be accelerated.

## Methods

### Fish strains and maintenance

The wild-type strain used in the screen was AB line. They were raised and staged as described [67]. Throughout the experiments, the embryos were incubated at 25°C. All experimental procedures on zebrafish embryos were approved by the Biological Research Centre, A*STAR, Singapore (BRC IACUC No. 080390).

### Identification of potential DUB genes in the zebrafish genome

For 5 types of DUB domains, PFAM [68] entries do exist and the corresponding hidden Markov models (HMMs) [69] were used for searches against the zebrafish proteome from ENSEMBL genome build database (originally with version v48 from February 2008 and, repeatedly, with later versions up to and including v53 from April 2009). The accessions of these five entries are: (i) UBQ-specific proteases (USPs) with the UBQ C-terminal hydrolase domain (UCH, PF0043), (ii) otubain proteases (OTUs) with the OTU-like cysteine protease domain (OTU, PF02338), (iii) UBQ C-terminal hydrolases (UCHs) with the UBQ C-terminal hydrolase family 1 domain (Peptidase_C12, PF1088), (iv) Machado-Joseph disease proteases (MJDs) with the Josephine domain (PF02099) and (v) JAMM motif metalloproteases (JAMMs) with the Mov34/MPN/PAD-1 family domain (PF01398).

Subsequently, the hits were blasted [70] against the zebrafish subset of the non-redundant protein sequence database for finding additional hits among sequences that were not included into the zebrafish genome build. Finally, blast searches against the human subset of the nrdb identified the closest human homologous proteins. Similar procedures were carried out for the PPPDE group and the WLMs (Wss1p-like proteases) group, where we relied on the published alignments [19] and on the PFAM entries PF08325 (WLM) and PF05903 (PPPDE). It should be noted that, depending on the zebrafish genome release and on the inclusion/removal of sequences into/from the non-redundant protein database, the *in silico *search results varied to some degree over time; the results presented here are an integration of the repeated searches.

### Morpholino (MO) sequence site selection and design

For each putative DUB gene found in the genome-wide *in silico *search, an individual morpholino was designed. First, we tried to find the translation initiation site (TIS) by mapping the protein sequence onto the genome or transcripts with BLAST http://blast.ncbi.nlm.nih.gov/Blast.cgi[70]. For proteins with obviously missing or incorrect N-termini, we inspected the respective genomic data and tried to find a TIS in agreement with sequences from other organisms. If this was not possible (and for sequences whose MOs for the TIS did not match the minimal MO quality criteria (see below)), we searched for exon/intron boundaries as a possible morpholino sequence site with the goal that the MO targeting site was located on or 5' to the UBQ protease domains.

Once the initiation site was selected, all potential upstream sequences for MO target oligos were processed with AMOD [71]. Antisense MOs were selected based on the guidelines from Gene Tools (reviewed by [23]). Basically, the MOs should have a GC content about 40-60%, less than 37% G content, and without any consecutive tri- or tetra-G nucleotide sequences. In addition, the selected MOs should minimize self or pair sequence homology. Furthermore, MOs that target 5'-UTR were generated for some selected DUB genes to test their specificity. Designed MOs are tabulated (Additional file 3).

### Morpholino specificity

Since the screen is a high-throughput knockdown analysis by using MO. Mistakes or non-specific knockdown might happen. However, we have tried our best to confirm the phenotypes by injecting the MOs and examined the phenotypes by at least two researchers. Besides, for those group I genes, we have designed a second MO that targets 5'-UTR region to further verify the knockdown phenotypes. In addition, we also used splicing MOs, which theoretically lead to un-spliced fragments encoding a stop codon within, and 5mis-match MOs of group IV DUBs to confirm that our knockdown results were specific and efficient. Sequences of MOs were listed in Additional file 3, and the result was tabulated in Additional file 10. Besides, the RT-PCR checks of splicing MOs were shown in Additional file 5.

### Expression construct generation and mRNA synthesis

*bmp4 *and *smad1 *were amplified with primers (for *bmp4*: 5'-CCGGATCCATGATTCCTGGTAATCGAATGCTGA-3' and 5'-CCCTCGAGTTAGCGGCAGCCACACCCCT-3'; for *smad1*: 5'-CCGAATTCATGAATGTCACCTCACTCTTTTCCT-3' and 5'-CCCTCGAGCTAGGACACTGAAGAAATGGGGTT-3') containing BamHI and XhoI; EcoRI and XhoI restriction sites, respectively, from full-length cDNA using *Pfu *DNA polymerase (Stratagene, La Jolla, CA) and ligated into pCS2+ to generate pCS2+*bmp4 *and pCS2+-*smad1 *expression constructs. All constructs cloned in the pCS2+ vectors were linearized by NotI. Capped RNA was prepared with the SP6 Message Machine kit (Ambion, Austin, TX) and finally dissolved in DEPC-treated water.

### Morpholino (MO) and mRNA injection

All MOs were purchased from Gene Tools, LLC (Philomath, OR), re-suspended in DEPC-treated water to make a 5 mM stock and stored at -20°C. Diluted MOs and/or mRNA (amount specified in Table 1 and Additional file 4) were injected into one- or two-cell stage embryos. Embryos from four different pairs of fish were used for each MO and/or mRNA injection.

### Screening procedure

Injected embryos (including control) were mainly scored at two time points (1 and 3 days postfertilization (dpf)), corresponding to the pharyngula period and the hatching period, respectively [67]. The screening examination was mainly based on the method described previously [22]. Briefly, embryos at early stages were examined for abnormalities in the shape and morphology of those early developing tissues or organs like eyes, brain, notochord, spinal cord and somites. Afterwards, in the latter stage, pigmentation and phenotypic changes in cardiovascular system and the fins were also examined. All phenotypes were observed in a dominant feature of injected embryos at a non-toxic dose by at least two screeners. Besides, the MO-induced effects were not accompanied with those non-specific effects caused by overdose MO treatment (unpublished observations). In addition, we also fixed embryos at 50-60% epiboly, 10-somite stage and 24 hpf for whole mount *in situ *hybridization (WISH).

### Whole mount in situ hybridization (WISH)

Plasmids that were used to make antisense mRNA probes have been published previously: *bmp4 *[72], *chd *[73], *eve1 *[36], *gata1 *[37], *gata2 *[37], *gsc *[39], *her4 *[29], *huC *[74], *myoD *[40], *pax2a *[43]. Single *in situ *hybridization was performed as described [75].

### RNA extraction, reverse transcription and quantitative real-time PCR

Embryos at 10-somite and prim-5 stages were collected. Their total RNA was extracted using TRIzol (Invitrogen, Carlsbad, CA). Purified sample RNA with a ratio of 1.8-2.0 at A260/A280 was used. Briefly, 0.5 μg of total RNA extracted was reversely transcribed by iScript cDNA synthesis kit (Bio-Rad, Hercules, CA). In case of real-time RT-PCR, PCRs were conducted using LightCycler 480 real-time PCR detection system using SYBR Green I Master (Roche, Basel, Switzerland). The copy number of the transcripts for each sample was calculated in reference to the parallel amplifications of known concentrations of the respective cloned PCR fragments. Standard curves were constructed and the amplification efficiencies were about 0.9-0.95. The data were then normalized using the expression levels of β-actin mRNA. The occurrence of primer-dimers and secondary products was inspected using melting curve analysis. Our data indicated that the amplification was specific. There was only one PCR product amplified for individual set of primers. Control amplification was done either without RT or without RNA. In the case of RT-PCR (underlined), embryos at prim-5 stage were used. PCRs were conducted by DNA Engine Dyad Peltier Thermal Cycler (Bio-Rad) using PCR Core Kit (Roche). Primers for *β-actin *(F: AGATCTGGCATCACACCTTC, R: TCACCAGAGTCCATCACGAT), *β-catenin2 *(F: AGGATCTGGACAACCAGGTG, R: GCACCATCACTGCAGCTTTA), *fgf8 *(F: GCAAGAAAAATGGTCTGGGA, R: TGCGTTTAGTCCGTCTGTTG), *her4 *(F: TGGCTCAAGAGTTCGTCAAG, R: AGTGGTCTGAGGATTGTCCA), *huC *(F: TCGAGTCCTGCAAATTGGTC, R: GTGAGGTGATGATCCTTCCA), *lefty1 *(F: CGCAAATTCACAAGAGGGAT, R: TCTCGGGGATTCTTGATGTC), *nodal1 *(F: GAGTGTGAGAGAAGCCCCTG, R: AGGTTCACTTCCACCACCAG), *otud4 *(F: GCGGTGCTTTGTCATTTACA, R: CTCTGAGAACGATCTTCTGG), *sprouty2 *(F: AGCAATGAGTACACGGAGGG, R: CACCTGCATTTCCCAAAAGT), *usp5 *(F: ATTTCGCTGCACCTTTGGTG, R: TGTTGGTCCTTCTTGATGTGG), *usp15 *(F: CATGCAGTGCGCGAGCGAAG, R: CTGAGAGCAGGCCGCTGTTG), *usp25 *(F: TTCATGCAGGAGCTTAGGCA, R: GCCAGGAAACGTCCATAAAA) and *wnt8a *(F: TGGTCGACTTGCTGTCAAAG, R: TCCATGTAGTCCCATGCTGA).

### Statistical analysis

All data are represented as the mean ± SEM. Statistical significance is tested by Student's t-test. Groups were considered significantly different if P < 0.05.

## List of abbreviations used

24 hpf: 24 hours after fertilization; BMP: bone morphogenetic protein; BRUCE: BIR repeat-containing Ubiquitin-conjugating enzyme; chd: chordin; DUBs: deubiquitylating enzymes; gsc: goosecoid; HA: hemagglutinin-tagged; JAMM: JAMM motif proteases; MIB: Mind bomb; MJD: Machado-Joseph disease protease; MO: morpholino; OTU: otubain protease; TGF-β: transforming growth factor β; TIS: translation initiation site; UBL: Ubiquitin-like; UBQ: Ubiquitin; UCH: Ubiquitin C-terminal hydrolase; UIM: Ubiquitin-interacting motif; USP: Ubiquitin-specific protease; WISH: whole mount *in situ *hybridization; WLMs: Wsslp-like proteases.

## Authors' contributions

WKFT, BE, FE and YJJ planned and designed the experiments. BE and FE contributed in the bioinformatics analysis of DUB genes. WKFT designed the MO and mRNA. WKFT, SHKH and QN performed the experiments. WKFT, BE, FE and YJJ wrote the manuscript. All authors read and approved the final manuscript.

## Supplementary Material

Additional file 1 Sequence Domain Architecture of zebrafish DUBs. Click here for file

Additional file 2 Reported DUB functions between 2005-2009. Click here for file

Additional file 3 **DUB genes in zebrafish**. The table lists the basic information of their names, Ensembl access/GenBank numbers and their MO sequences. Click here for file

Additional file 4 Early developmental phenotypes of zebrafish after DUBs MO knockdowns. Click here for file

Additional file 5 This figure shows the PCR products of group IV DUBs after injecting splicing MOs and the expression levels of *huC *and *her4 *in morphants of group I and selected group II DUB genes by using RT-PCR. Click here for file

Additional file 6 This figure shows the morphologies of dorsalized (C1-C5) and ventralized (V1-V4) embryos after microinjections at 24-30 hpf. Click here for file

Additional file 7 This figure presents the lateral view of *in situ *hybridization data of ventralized markers (*bmp4, eve1, gata2*) at 50-60% epiboly morphants. Click here for file

Additional file 8 This figure shows the results of *in situ *hybridization experiments, using *gsc*, *pax2a + myoD *probe at 50-60% epiboly and prim-5 stage. Click here for file

Additional file 9 This figure shows the mRNA expression level of different signaling genes (*β-catenin2, fgf8, lefty1, nodal1, sprouty2 *and *wnt8a*) at 10-somite stage of group IV DUB morphants. Click here for file

Additional file 10 Frequency of dorsalized phenotypes of group IV MOs injection studies. Click here for file
