# Supplementary material for: Genome-wide loss-of-function analysis of deubiquitylating enzymes for zebrafish development
Source: BMC Genomics. 2009 Dec 30;10:637. doi: 10.1186/1471-2164-10-637 (PMC2809080; doi:10.1186/1471-2164-10-637)
Supplement: Additional file 1 — Sequence Domain Architecture of zebrafish DUBs. [file 1471-2164-10-637-S1.PDF]

Additional file 1

Title: Sequence Domain Architecture of zebrafish DUBs

File format: PDF

A. USP: ***UCH (PF0043)***, ubiquitin-specific protease family

Gene [chromosome]	Protein Accession	Sequence Domain Architecture
cylda [7]	XP_684817.3	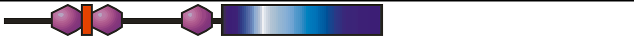
cyldb [11]	XP_692599.2	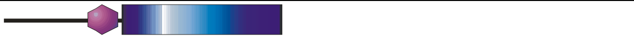
usp1 [6]	NP_955873.1	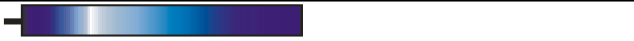
usp2a [15]	NP_001008574.1	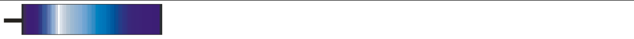
usp2b [5]	XP_001337596.1	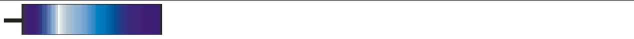
usp3 [25]	Ensemble[1]	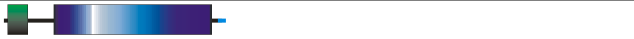
usp4 [6]	XP_001341961.2	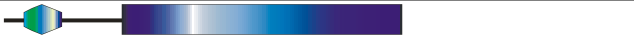
usp5 [16]	NP_999920.2	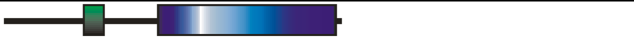
usp7 [3]	XP_691215.3	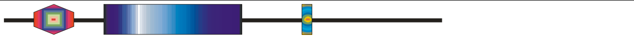
usp8 [18]	XP_693811.2	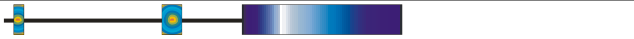
usp9 [9]	NP_001070917.1	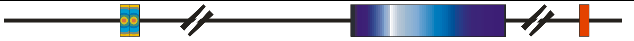
usp10 [7]	XP_685621.3	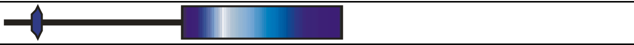
usp11 [8]	XP_696751.2	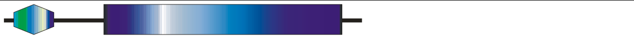
usp12a [24]	NP_001077025.1	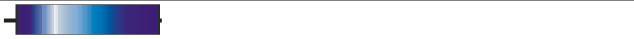
usp12b [7]	XP_688359.2	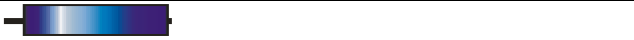
usp13 [6]	NP_001091856.1	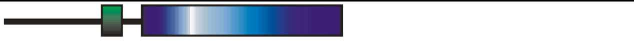
usp14 [18]	NP_956267.1	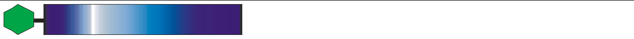
usp15 [4]	XP_684202.2	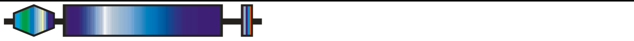
usp16 [15]	A8HAL1	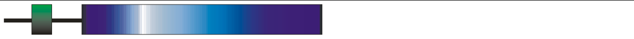
usp18 [4]	XP_001332402.1	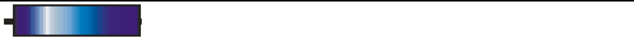
usp19 [23]	XP_689922.3	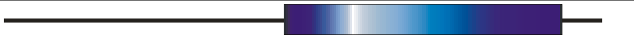
usp20 [5]	NP_957281.2	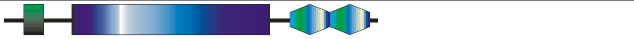
usp21 [23]	XP_692003.2	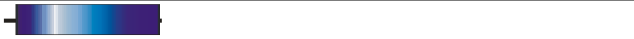
usp22 [12]	AAI46619.1	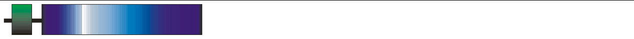
usp24 [5]	XP_001920230.1	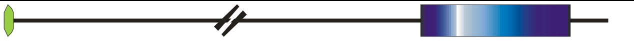
usp25 [10]	NP_001001886.1	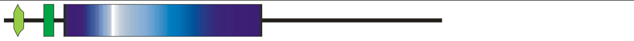
usp28 [21]	XP_001920096.1	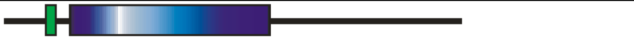
usp30 [5]	XP_687498.3	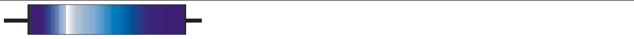
usp31 [3]	XP_692912.2	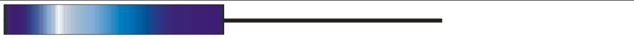
usp32 [15]	XP_686236.3	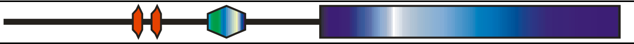
usp33 [2]	NP_998392.1	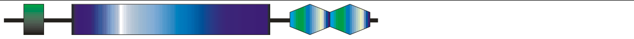
usp34 [18]	XP_001921595.1	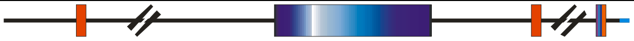
usp36 [3]	XP_688241.3	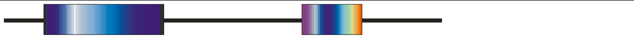
usp37 [9]	NP_001070811.1	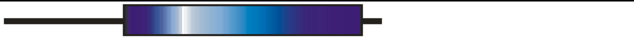
usp38 [1]	XP_001919108.1	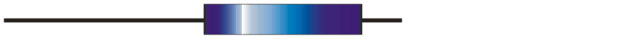

usp39 [5]	NP_001073539.1	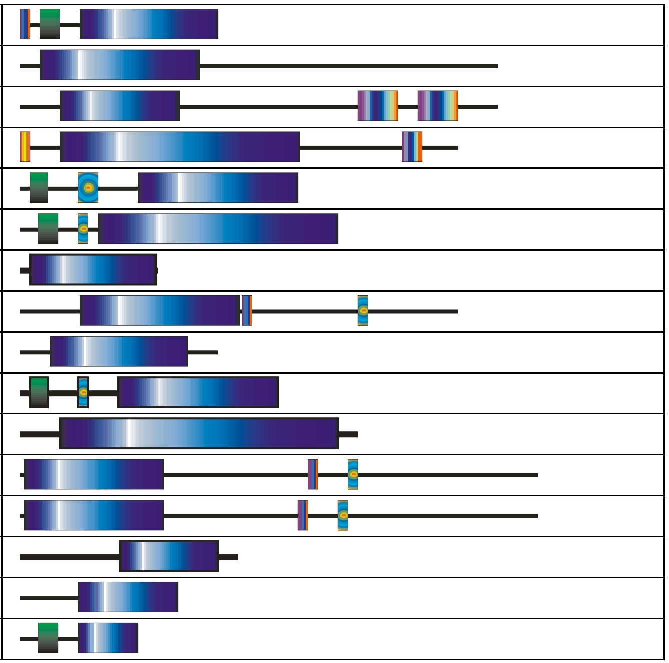
usp40 [6]	XP_001921353.1	
usp42 [12]	XP_693784.3	
usp43 [6]	NP_001082871.1	
usp44 [4]	NP_956551.1	
usp45 [16]	XP_692456.3	
usp46 [20]	XP_001923229.1	
usp47 [Un]	Ensemble[2]	
usp48 [11]	NP_001019581.1	
usp49 [22]	NP_001038361.1	
usp53 [7]	XP_001331966.2	
usp54a [13]	XP_692367.3	
usp54b [12]	XP_694338.3	
unclassified (1) [2]	XP_693929.1	
unclassified (2) [13]	XP_001334389.1	
unclassified (3) [16]	XP_001336666.1	

500 amino acids	
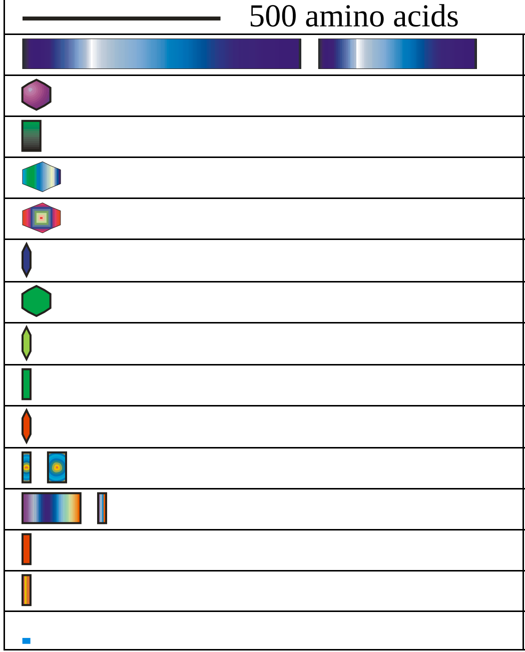	*UCH*
	CAP_Gly (PF01302)
	Zf-UBP (PF02148)
	DUSP (SM00695)
	MATH (PF00917)
	PAM2 (PF07145)
	UBQ (SM00213)
	UBA (PF00627)
	UIM (PF02809)
	EFh (SM00054)
	Coiled-Coil region
	Mixed charge cluster
	Negative charge cluster
	Positive charge cluster
	PTS1 Signal

Ensemble[1] = ENSDART00000078908,
Ensemble[2] = ENSDART00000074614

B. OTU: *OTU* (PF02338)

Gene [chromosome]	Protein Accession	Sequence Domain Architecture
otub1 [7]	NP_001002500.1	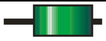
otub1l [21]	NP_001002411.1	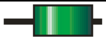
otud3 [23]	NP_998087.1	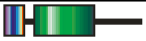
otud4 [21]	NP_001027008.1	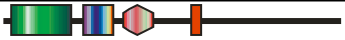
otud5a [8]	Q08BW0	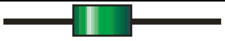
otud5b [11]	NP_001003540.1	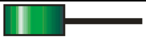
otud6b [19]	Q7ZV00	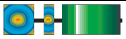
otud7a [25]	XP_687887.2	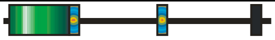
otud7b [16]	NP_001071212.1	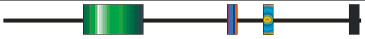
parp11 [23]	XP_001923008.1	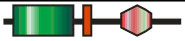
tnfaip3 [13]	XP_692922.3	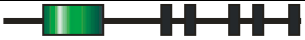
vcpip1 [2]	XP_699171.1	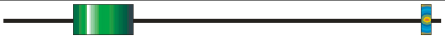
yod1 [11]	Q567B1	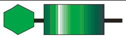
zranb1a [12]	XP_001919972.1	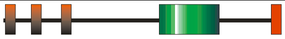
zranb1b [17]	NP_001071236.1	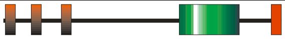

500 amino acids	
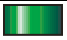	*OTU*
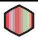	*Tudor* (SM00333)
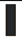	*Zf-A20* (PF01754)
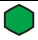	*UBQ* (SM00213)
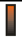	*Zf-RanBP* (PF00641)
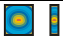	Coiled-Coil region
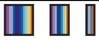	Mixed charge cluster
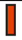	Negative charge cluster

C. UCH: *Peptidase_C12* (PF01088), Ubiquitin carboxyl-terminal hydrolase, family 1

Gene [chromosome]	Protein Accession	Sequence Domain Architecture
bap1 [22]	XP_687254.2	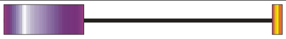
uchl1 [1]	Q6YI49	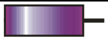
uchl3 [9]	Q504C0	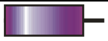
uchl5 [22]	Q6NWL6	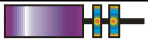

500 amino acids	
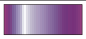	*Peptidase_C12*
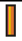	Positive charge cluster
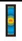	Coiled-Coil region

D. MJD: ***Josephin*** **(PF02099)**, Machado-Joseph disease (MJD)

Gene [chromosome]	Protein Accession	Sequence Domain Architecture
atxn3 [15]	NP_957398.1	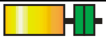
josd1 [12]	NP_001070063.1	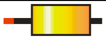
josd2 [16]	NP_956445.1	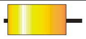

500 amino acids	
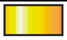	*Josephin*
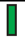	*UIM* (PF02809)
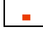	Myristoylation signal

E. JAMM: ***Mov34*** **(PF01398)/*JAB_MPN*** **(SM00232),** Mov34/MPN/PAD-1 family

Gene [chromosome]	Protein Accession	Sequence Domain Architecture
brcc3 [22]	XP_001342274.1	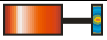
cops5 [24]	Q6PC30	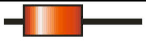
cops6 [14]	NP_001017768.1	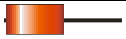
eif3f [1]	XP_685399.1	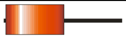
eif3ha [16]	NP_001003763.1	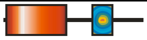
eif3hb [19]	NP_001030341.1	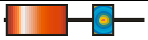
mpnd [8]	Q08CH3	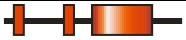
mysm1 [20]	XP_689723.2	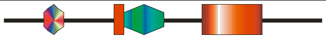
prpf8 [15]	NP_957270.2	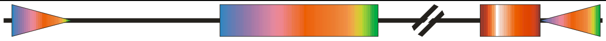
psmd7 [7]	NP_956083.1	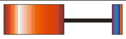
psmd14 [11]	NP_001077042.1	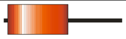
stambpa [10]	Q6TH47	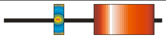
stambpb [8]	NP_001032659.1	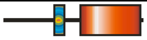
stambpl1 [12]	XP_699129.2	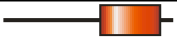

500 amino acids	
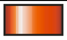	*Mov34/JAB_MPN*
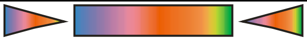	*PRO8:* (PF08082), (PF08083), (PF08084)
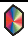	*Myb_DNA_binding* (PF00249)
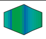	*SWIRM* (PF04433)
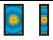	Coiled-Coil region
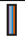	Mixed charge cluster
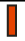	Negative charge cluster

<u>F. PPPDE:</u> *DUF862* (PF05903), PPPDE putative peptidase domain

**Gene [chromosome]**	**Protein Accession**	**Sequence Domain Architecture**
pppde1 [13]	Q6DC39	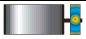
LOC794838 [13]	XP_001923966.1	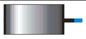
pppde2a [3]	NP_956994.1	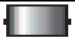
pppde2b [12]	NP_956502.1	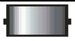

500 amino acids	
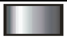	*DUF862*
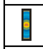	Coiled-Coil region
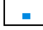	PTS1 signal
