# Supplementary material for: Genome-wide loss-of-function analysis of deubiquitylating enzymes for zebrafish development
Source: BMC Genomics. 2009 Dec 30;10:637. doi: 10.1186/1471-2164-10-637 (PMC2809080; doi:10.1186/1471-2164-10-637)

Additional file 5

Title: RT-PCR of group IV zebrafish DUB genes in their corresponding morphants and *huC* and/or *her4* expression in group I and selected group II morphants

File format: PDF

Description: Panel A proved the specificity of group IV DUB MOs by using splicing MOs. Up-shift of PCR products (marked as red star), which was due to the incomplete splicing processes, was seen for all group IV splicing MOs. Panel B further confirmed the *in situ* results of increased *huC* and decreased *her4* expression in group I morphants by RT-PCR. Panel C showed decreased *huC* expression after RT-PCR in selected group II morphants. ß-actin was used for normalization. Values in white indicated the relative quantified data by Sigma Scan.

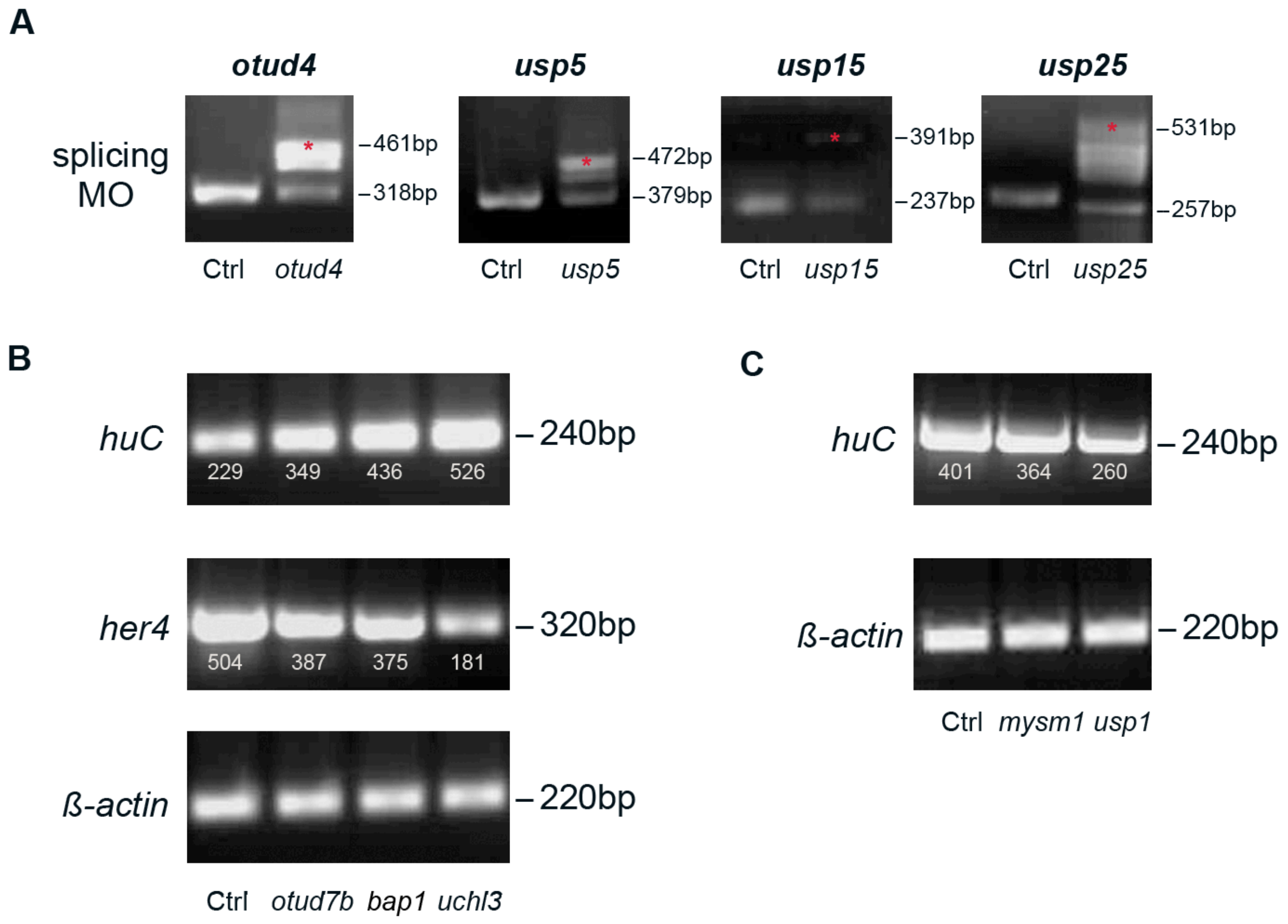

Supplement: Additional file 5 — This figure shows the PCR products of group IV DUBs after injecting splicing MOs and the expression levels of huC and her4 in morphants of group I and selected group II DUB genes by using RT-PCR. [file 1471-2164-10-637-S5.PDF]
