# Supplementary material for: Genome-wide loss-of-function analysis of deubiquitylating enzymes for zebrafish development
Source: BMC Genomics. 2009 Dec 30;10:637. doi: 10.1186/1471-2164-10-637 (PMC2809080; doi:10.1186/1471-2164-10-637)

Additional file 6

Title: Phenotype of dorsalized (C1-C5) and ventralized (V1-V4) embryos at 24-30 hpf

File format: PDF

Description: The phenotypic description is based on Mullins *et al.* (1996) and Kishimoto *et al.* (1997). Dorsalized C1 phenotype features a partial loss of ventral tail fin; C2 shows a loss of ventral fin; C3 is characterized by normal trunk but winding up tail; C4 has an abnormal body axis that is wound up in a snail shell-like fashion with shorten tail and C5 is the strongest dorsalized phenotype that is characterized by football-shaped embryos after gastrulation. Besides, there is a loss of tail development in the embryos. Ventralized V1 phenotype has smaller head and eyes; V2 features an expansion of posterior somite with a slight increase of blood island; V3 shows little or loss of head structure with expanded posterior somites and enlarged blood island and V4 embryo is characterized by complete abnormal anterior parts with identified somites.

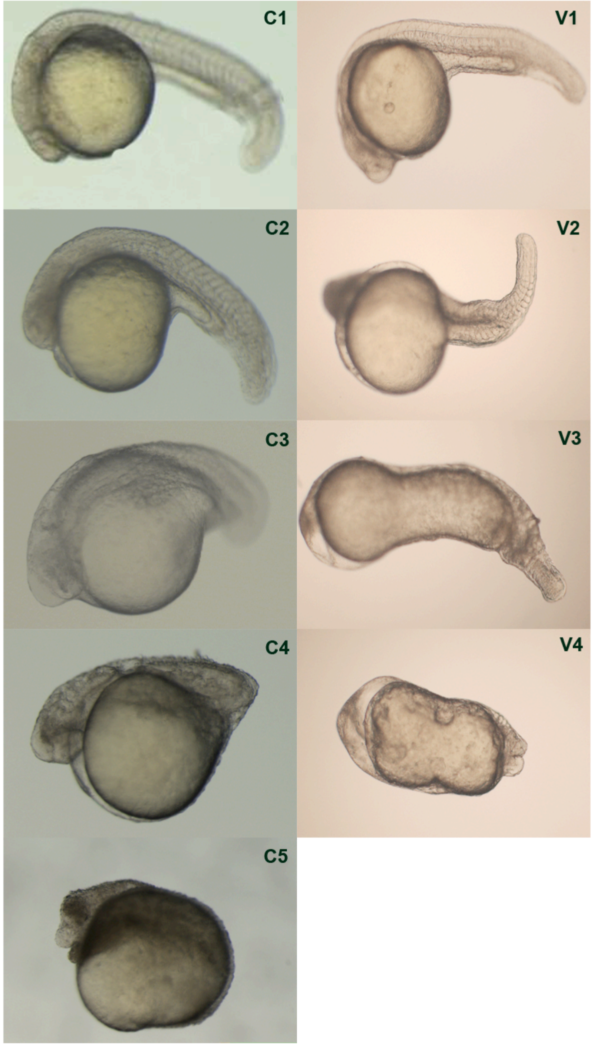

Supplement: Additional file 6 — This figure shows the morphologies of dorsalized (C1-C5) and ventralized (V1-V4) embryos after microinjections at 24-30 hpf. [file 1471-2164-10-637-S6.PDF]
