# Supplementary material for: Genome-wide loss-of-function analysis of deubiquitylating enzymes for zebrafish development
Source: BMC Genomics. 2009 Dec 30;10:637. doi: 10.1186/1471-2164-10-637 (PMC2809080; doi:10.1186/1471-2164-10-637)

Additional file 7

Title: Lateral view of ventral markers (*bmp4, eve1, gata2*) expression in morphants of group IV zebrafish DUBs at 50-60% epiboly

File format: PDF

Description: In addition to the animal pole view shown in Figure 3, expression of ventral markers (*bmp4*, *eve1* and *gata2*) at 50-60% epiboly stage was also shown in lateral views, animal pole towards the left. *otud4* (B, G, L), *usp5* (C, H, M) and *usp25* (E, J, O) morphants showed narrower expression pattern for ventral markers (A-O). *usp15* showed similar expression pattern (D, I, N) with control (A, F, K). Red dot lines indicated the normal expression margin of ventral markers in wild-type embryos.

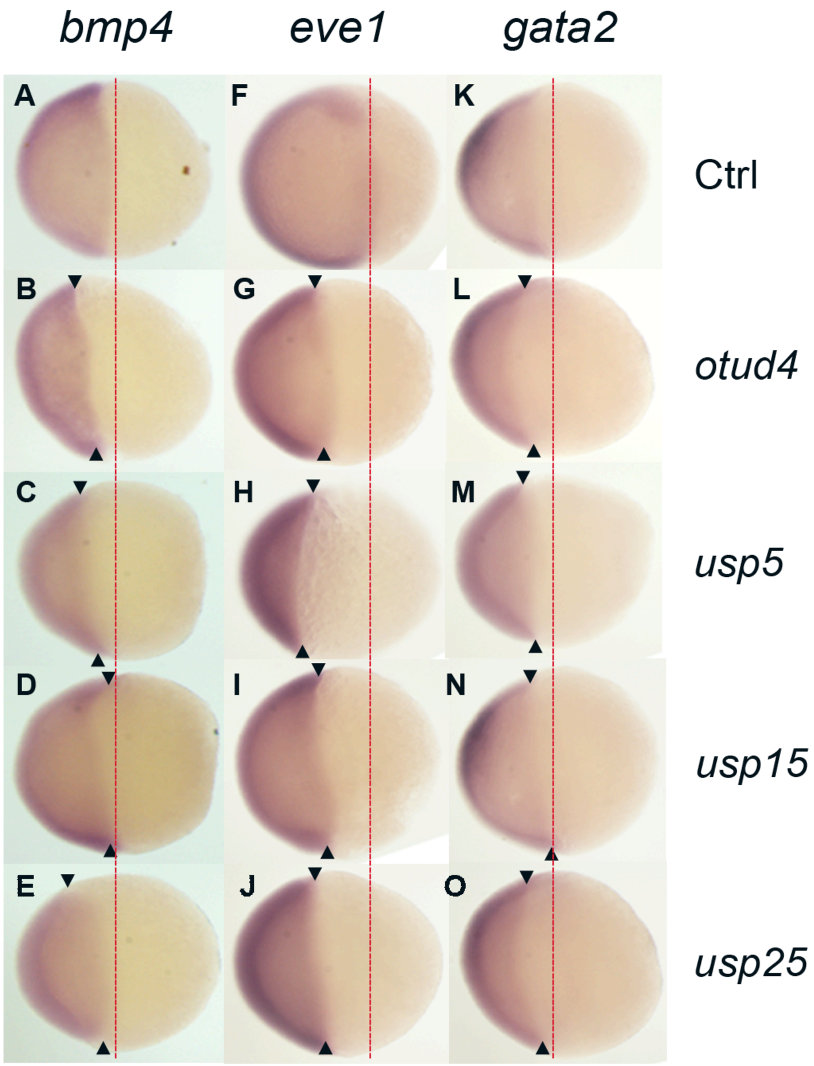

Supplement: Additional file 7 — This figure presents the lateral view of in situ hybridization data of ventralized markers (bmp4, eve1, gata2) at 50-60% epiboly morphants. [file 1471-2164-10-637-S7.PDF]
