# Supplementary material for: Genome-wide loss-of-function analysis of deubiquitylating enzymes for zebrafish development
Source: BMC Genomics. 2009 Dec 30;10:637. doi: 10.1186/1471-2164-10-637 (PMC2809080; doi:10.1186/1471-2164-10-637)

Additional file 8

Title: *In situ* hybridization results of selected genes at different stages of embryonic development after overexpression and knockdown experiment

File format: PDF

Description: Additional *in situ* hybridization experiments at different stages had been preformed to confirm the dorsalized or ventralized phenotypes after being examined by eyes. Panels A to C showed the *bmp4* mRNA expression pattern at prim-5. Noted that there is a decrease of mRNA expression in the head region (arrows) for dorsalized embryos (A) when compared to control (B). Ventralized embryos (C) are featured with the ectopic expression at the tip of the yolk extension as well as the distal border of the expanded blood island (arrows). Panels D to I, animal pole view, dorsal towards the right; J to O, dorsal view, showed the *gsc* mRNA expression pattern at 50-60% epiboly. Different degree of expanded expression patterns were found in the dorsalized embryos (D, E, J, K). In contrast, restricted patterns were found in the ventralized embryos (G-I, M-O) when compared to control (F, L). Panels P to A' showed the mRNA expression pattern of *pax2a* and *myoD* at prim-5 stage. R and X were the control. Noted that dorsalized embryos were characterized by shortened and uneven (P, V) and/or laterally expanded (Q, W) *myoD* expression. In ventralized embryos (S-U, Y-A'), *myoD* expression is stronger at the tail end. Abnormal tail development was indicated by fused *myoD* patterning. P to U, lateral views, anterior to the left; V to A', dorsal view.

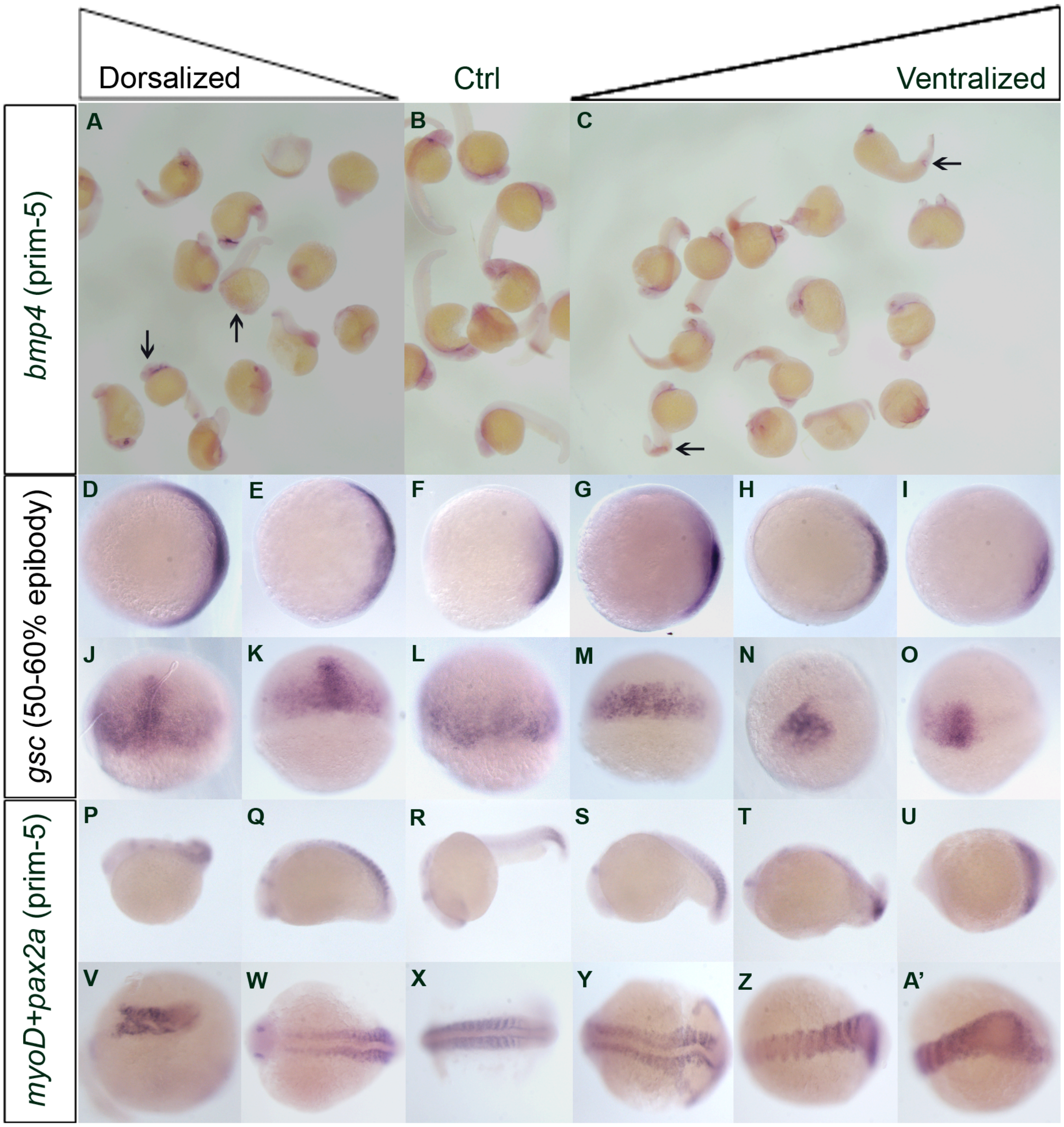

Supplement: Additional file 8 — This figure shows the results of in situ hybridization experiments, using gsc, pax2a + myoD probe at 50-60% epiboly and prim-5 stage. [file 1471-2164-10-637-S8.PDF]
