# Supplementary material for: Genome-wide loss-of-function analysis of deubiquitylating enzymes for zebrafish development
Source: BMC Genomics. 2009 Dec 30;10:637. doi: 10.1186/1471-2164-10-637 (PMC2809080; doi:10.1186/1471-2164-10-637)

Additional file 9

Title: Quantitative real-time PCR analysis of genes in different signaling at 10-somite stage after knockdown of group IV DUB genes

File format: PDF

Description: Increase in mRNA expression was found in *fgf8* (A) while its antagonist, *sprouty2* (*spy2*) was decreased (B). In addition, mRNA expression of *nodal1* (*ndl1*) was increased (C), while its antagonist, *lefty1* (*lft1*) was decreased (D). There was no significant change in the *wnt8* mRNA expression (E) but its transcriptional effector, *β-catenin2* showed an increased mRNA expression (F). Increased mRNA expression of *nodal* and *β-catenin2* leads to an increase of *fgf* signal that could stimulate the Bmp inhibitor, Chordin; and, therefore, inhibits Smad in the Bmp pathway that resulted in dorsalized phenotypes. Error bars represent the SEM of three technical replicates. * P<0.05 compared with the control.

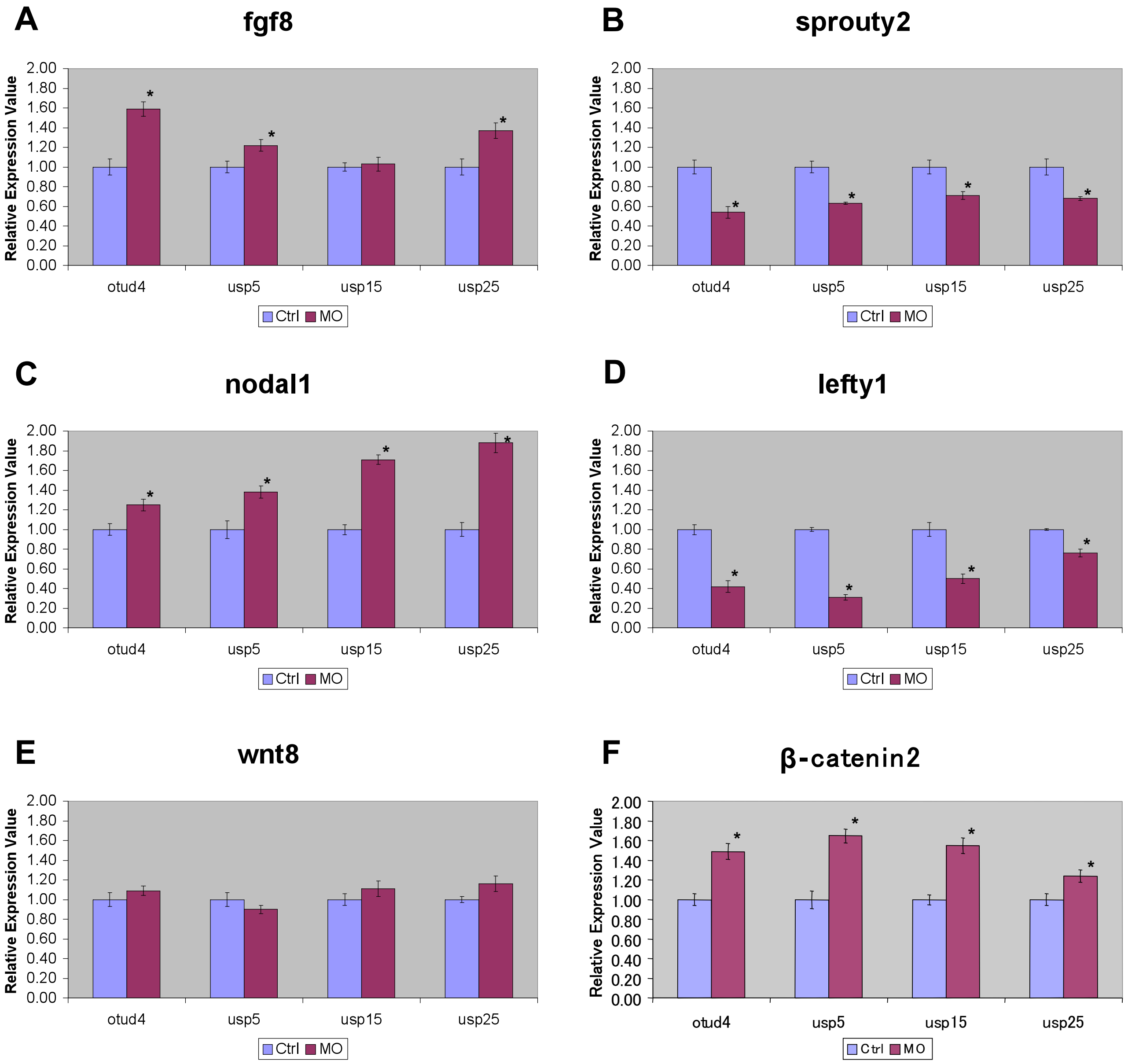

Supplement: Additional file 9 — This figure shows the mRNA expression level of different signaling genes (β-catenin2, fgf8, lefty1, nodal1, sprouty2 and wnt8a) at 10-somite stage of group IV DUB morphants. [file 1471-2164-10-637-S9.PDF]
