# Supplementary material for: Genome-wide loss-of-function analysis of deubiquitylating enzymes for zebrafish development
Source: BMC Genomics. 2009 Dec 30;10:637. doi: 10.1186/1471-2164-10-637 (PMC2809080; doi:10.1186/1471-2164-10-637)
Supplement: Additional file 10 — Frequency of dorsalized phenotypes of group IV MOs injection studies. [file 1471-2164-10-637-S10.PDF]

Additional file 10

Title: Frequency of dorsalized phenotypes of group IV MOs injection studies
File format: PDF

MO	pmol	n ep	n	C5 %	C4 %	C3 %	C2 %	C1 %	WT %	DV value
otud4-MO1	0.5	3	104	8	34	28	24	6		3.14
usp5-MO1	2.0	3	101		12	42	30	16		2.50
usp15-MO1	0.5	3	127		5	21	53	21		2.10
usp25-MO1	2.0	3	185	10	28	38	14	10		3.14
otud4-MO2	0.5	2	80	2	22	40	24	12		2.78
usp5-MO2	2.0	2	73	6	19	38	27	10		2.84
usp15-MO2	2.0	2	66		7	29	43	21		2.22
usp25-MO2	2.0	2	72	5	16	30	22	27		2.50
S-otud4	1.0	2	93		30	39	25	6		2.93
S-usp5	2.5	2	96	6	25	37	18	14		2.91
S-usp15	2.5	2	86		2	33	31	34		2.03
S-usp25	2.5	2	90	8	23	33	17	19		2.84
M-otud4	2.5	2	61						100	0.00
M-usp5	2.5	2	66						100	0.00
M-usp15	2.5	2	58						100	0.00
M-usp25	2.5	2	70						100	0.00

The frequencies of changing phenotypes after injecting group IV MOs were marked in the table. C1-C5 phenotypes represented dorsalized phenotypes as described in [30,31]; The dorsal-ventral values were listed in the table that were calculated as follows: sum of (frequency of phenotypes * [C5: 5; C4: 4; C3: 3; C2: 2; C1: 1; Wt: 0; V1: -1; V2: -2; V3: -3; V4: -4]). The highest DV value is 5, which indicated 100% C5 phenotypes. Photos of phenotypes were shown in Additional file 6. MO1 is targeting 5'-ATG region; MO2 is targeting 5'-UTR region; S represents splicing MO; and M for 5mis-match MO.

Abbreviations: n ep, number of experiments; n, number of scored embryos; DV value, dorsal-ventral value.
